# Automation of environmental ELISAs

**DOI:** 10.1155/S146392469400026X

**Published:** 1994

**Authors:** C. B. Shumate, J. E. Johnson, D. A. Fitzpatrick, C. Charan

**Affiliations:** 1 Hamilton Company 4970 Energy Way Reno NV 89502 USA; 2 Quantix Systems 2611 Branch Pike Cinnaminson NJ 08077 USA; 3 Midwest Research Institute 625-B Clyde Avenue Mountain View CA 94043 USA

## Abstract

ELISAs for pesticides and herbicides in environmental and agricultural samples are becoming very important in screening applications [1-3]. Traditional chromatographic methods are expensive and results need long turnaround times, making them incompatible with rapid on-site decision making. ELISA methods have been shown to meet or exceed the performance of gas chromatography—they offer rapid low-cost analysis, thereby increasing the frequency of sampling and enhancing data quality. Automated ELISA workstations allow the full benefit of these kits to be realized. Sample preparation, reagent pipetting, incubation, and photometric evaluation can be performed without user intervention. Reliability is increased through the elimination of operator error, better accuracy and precision, and often higher speed. Much larger batch sizes are possible and these systems can provide sample tracking with report generation for documentation requirements. In this paper the manual procedures and ELISA methods are compared and some critical aspects of automating these ELISA kits are discussed.


Journal of Automatic Chemistry, Vol. 16, No. 6 (November-December 1994), pp. 211-218

### Automation of environmental ELISAs

**C. B. Shumate, J. E. Johnson**
*Hamilton Company, 4970 Energy Way, Reno, NV 89502, USA*
**D. A. Fitzpatrick**
*Quantix Systems, 2611 Branch Pike, Cinnaminson, NJ 08077, USA*
**and C. Charan**
*Midwest Research Institute, 625-B Clyde Avenue, Mountain View, CA 94043, USA*

*ELISAs for pesticides and herbicides in environmental and agricultural samples are becoming very important in screening applications [1–3]. Traditional chromatographic methods are expensive and results need long turnaround times, making them incompatible with rapid on-site decision making. ELISA methods have been shown to meet or exceed the performance of gas chromatography—they offer rapid low-cost analysis, thereby increasing the frequency of sampling and enhancing data quality. Automated ELISA workstations allow the full benefit of these kits to be realized. Sample preparation, reagent pipetting, incubation, and photometric evaluation can be performed without user intervention. Reliability is increased through the elimination of operator error, better accuracy and precision, and often higher speed. Much larger batch sizes are possible and these systems can provide sample tracking with report generation for documentation requirements. In this paper the manual procedures and ELISA methods are compared and some critical aspects of automating these ELISA kits are discussed.*

### Introduction

The Ohmicron RaPID assay for atrazine has been automated with the Hamilton Microlab 2200. This is a magnetic particle-based assay performed in test tubes. Modifications to the instrument include a pneumatic rack system and dual lumen probe with vacuum capabilities for tube washing. The Quantix plate-based assays for alachlor and metalochlor were pipetted on the Hamilton Microlab ATplus. Spiked soil extracts in methanol were automatically diluted and pipetted into both alachlor and metalochlor test wells contained in the same plate frame. Several dilutions can be programmed to ensure that results fall within calibration range. The alachlor assay was pipetted on the ATplus and transferred to the Hamilton Microlab F.A.M.E. for incubation, reagent addition, plate washing, and photometric evaluation.

### Experimental

### *Ohmicron RaPID® test tube based assays on the Microlab® 2200*

The liquid handling system used for the Ohmicron RaPID Atrazine assay (Ohmicron Corp., cat. No. A00071, Newtown, PA, USA) is based on a Hamilton Microlab 2200 automated pipettor with minor modifications (see figure 1). The Microlab 2200 is a cartesian co-ordinate robotic liquid handling station with a work envelope of 14 in (D) × 32 in (L) × 6 in (H). The three-axis drive system comprises closed loop digitial DC servo motors. A natural language user interface, FLEXPREP version 6.1.1, which resembles BASIC, is used to program probe movement, syringe and valve position, and accessory control.

A dual probe option (PN 36950) was employed with two syringe drives (see figure 2). A Teflon coated tapered probe (0·055 in i.d. tapered to 0·022 in) for precise sample and conjugate transfers was plumbed to a 100 µl syringe and an untapered probe (0·055 in i.d.) was connected to a 10 ml syringe which allowed multiple aliquots of reagents to be dispensed quickly. A three-way valve on the large syringe allowed a vacuum connection for tube washing. The two probes were separated by 3 mm, with the untapered probe extending 1 mm lower than the tapered probe. This allowed the untapered probe to touch the bottom of the assay tubes for complete evacuation during tube washing. The capacitive liquid level detection circuitry would trigger on the untapered probe, but still allowed the tapered probe to remain below the liquid surface after detection. A vacuum pump (Air Cadet, Cole-Parmer, cat. No. 7530-40, 20 in Hg) was connected through a 2 l side arm flask to the top port of the valve on the 10 ml syringe. The pump ran continuously during the assay. A Hamilton four port solvent selection Modular Valve Positioner (MVP) was used to select between wash solution, magnetic beads, chromogen and stop solution. The magnetic beads were of the order of 1 µm and remained in homogeneous solution for over 1 h after mixing—this feature was crucial to the success of automating this assay. Other solid phase based assays using latex spheres can be kept continuously mixed by a magnetic stirrer; an option obviously unavailable when using magnetic particles. The chromogen required a binary mixture to be made fresh before use.

A custom assay tube rack system was constructed using two pneumatic cylinders (Bimba Co., model 021-R, Monee, IL, USA) to raise and lower the assay tubes into the magnetic base for washing. Compressed air, regulated to 15 psi, was delivered to the cylinders through another MVP with two ports. These MVPs are controlled via FLEXPREP through a serial communication daisy chain from the 2200.

Sample tubes were located on one side of the deck and two 60-tube assay racks were located on the other. Controls and calibrators could be located within the sample rack or in a reagent rack located in the center of the deck. The kit's original Conjugate container was placed between the sample rack and assay racks. A wash station allows the probes to be washed by an internal flushing and external overflow.

The sequence of steps is as follows:

(1) Aspirate a 10 µl air gap followed by 350 µl of conjugate (100 µl overfill) and 200 µl of sample using 1000 µl syringe.

0142-0453/94 $10.00 © 1994 Taylor & Francis Ltd.

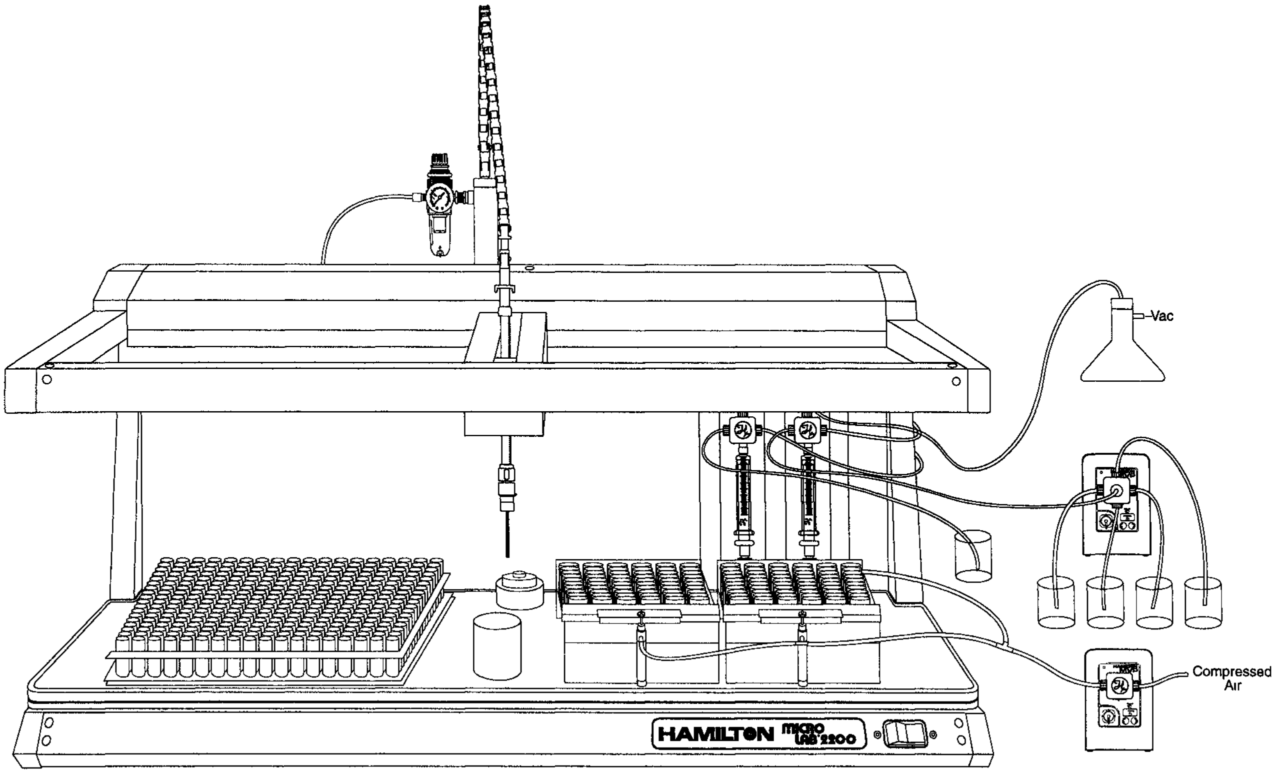

*Figure 1. Hamilton Microlab 2200 with Ohmicron RaPID assay configuration. Sample tubes are located on the left, reagents and wash station in the centre, and two 60-position assay racks with magnetic bases on the right. Note pneumatic cylinders for movement of assay tubes into magnetic base.*

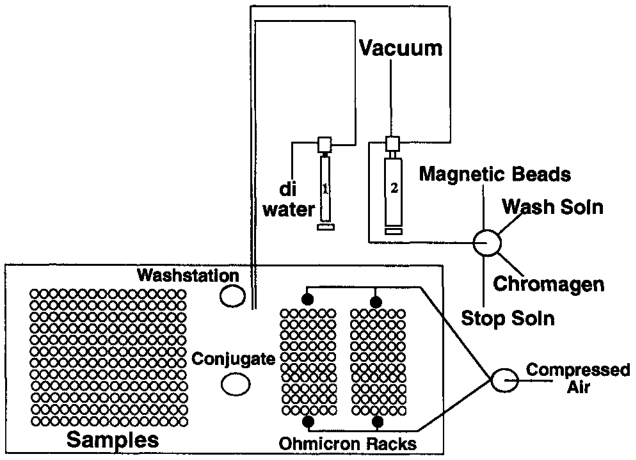

*Figure 2. Microlab 2200 system diagram for Ohmicron RaPID assay.*

(2) Dispense 450 µl to assay tube.
(3) Wash with 1000 µl of system fluid (air gap and overfill of conjugate are dispensed to the wash station first).
(4) Repeat for all samples.
(5) Turn solvent select MVP to position 2, magnetic particles, and reprime 10 ml syringe.
(6) Aspirate 10 ml of magnetic particles using 10 ml syringe.
(7) Dispense 20 aliquots of 500 µl to first 20 assay tubes.
(8) Repeat for remaining tubes.
(9) Incubate for 15 min.
(10) Activate pneumatic cylinders with second MVP.
(11) Wait 2 min for particles to capture against walls.
(12) Turn on vacuum through untapered probe and evacuate all assay tubes.
(13) Turn solvent select MVP to wash solution and reprime.
(14) Aspirate 10 ml of wash solution using 10 ml syringe.
(15) Dispense 10 aliquots of 1 ml to first 10 tubes.
(16) Repeat for remaining tubes.
(17) Repeat evacuation and wash solution dispense ending with evacuation.
(18) Turn off pressure to pneumatic cylinders to raise tube rack away from magnetic base.
(19) Turn solvent select MVP to Chromogen and reprime.
(20) Dispense 500 µl of Chromogen to all assay tubes.
(21) Incubate for 20 min.
(22) Turn solvent select MVP to Stop solution and reprime.
(23) Dispense 500 µl of Stop solution to all assay tubes.
(24) Manually read all calibrators, controls, and samples.
(25) Turn solvent select MVP to wash solution and reprime system.

An example of the FLEXPREP instruction set required to direct the method is shown in figure 3.

The supercritical fluid extraction and immunoassay of soil samples has been reported previously [4]. Quadruplicate 3 g samples of topsoil and clay were spiked with atrazine (ChemService, Westchester, PA, USA) at 10, 50 and 500 ppb. A matrix blank was prepared with 3 g of topsoil and a system blank contained only glass wool. The samples

```
10 PRINT "HOW MANY SAMPLES ARE YOU RUNNING?".
20 INPUT N.
30 COMMENT ***BEGIN SAMPLE AND CONJUGATE ADDITION****.
40 FOR I=1 TO N.
50 ASPIRATE 10 OF AIR.
60 ASPIRATE 350 OF CONJUGATE.
70 ASPIRATE 200 OF P:SAMPLES(I).
80 DISPENSE 450 TO S:RACKUP(I).
90 WASH 1000.
100 NEXT I.
110 COMMENT**BEGIN MAGNETIC PARTICLE ADDITION*****.
120 FOR I=0 TO N-1 STEP 20.
130 ASPIRATE 10000 OF DILUENT.
140 FOR J=I TO I+19.
150 DISPENSE 500 TO S:RACKUP(J+1).
160 NEXT J.
170 NEXT I.
180 COMMENT***MAGNETIC PARTICLE INCUBATION*****.
190 WAIT 900.
200 COMMENT***PULL RACK DOWN INTO MAGNETIC BASE****.
210 ACTUATE DEVdLP02R.
220 WAIT 120.
230 COMMENT***TURN ON VACUUM FOR EVACUATION***.
240 ACTUATE DEVbCWR.
250 FOR I=1 TO N.
260 MOVE TO S:RACKDOWN(I) AT ZMAX.
270 NEXT I.
280 COMMENT***TURN SOLVENT SELECT MVP TO WASH SOLUTION***.
290 ACTUATE DEVcLP02R.
300 COMMENT***REPRIME WITH WASH SOLUTION***.
310 WASH 6000.
320 COMMENT***PERFORM WASH TWICE***.
330 FOR Z=1 TO 2.
340 FOR I=0 TO N-1 STEP 10.
350 ASPIRATE 10000 OF DILUENT.
360 FOR J=I TO I+9.
370 DISPENSE 1000 TO S:RACKDOWN(J+1).
380 NEXT J.
390 NEXT I.
400 COMMENT***TURN ON VACUUM FOR EVACUATION****.
410 ACTUATE DEVbCWR.
420 FOR I=1 TO N.
430 MOVE TO S:RACKDOWN(I) AT ZMAX.
440 NEXT I.
450 WASH 2000.
460 NEXT Z.
470 COMMENT***TURN PRESSURE OFF TO RAISE RACK***.
480 ACTUATE DEVdLP11R.
490 COMMENT***TURN SOLVENT SELECT MVP TO COLOR REAGENT***.
500 ACTUATE DEVcLP03R.
510 COMMENT***REPRIME WITH COLOR REAGENT***.
520 WASH 6000.
530 COMMENT***BEGIN COLOR REAGENT ADDITION***.
540 TIMER 1200.
550 FOR I=0 TO N-1 STEP 20.
560 ASPIRATE 10000 OF DILUENT.
570 FOR J=I TO I+19.
580 DISPENSE 500 TO S:RACKUP(J+1).
590 NEXT J.
600 NEXT I.
610 COMMENT***TURN SOLVENT SELECT MVP TO STOP SOLUTION***.
620 ACTUATE DEVcLP04R.
630 COMMENT***REPRIME WITH STOP SOLUTION***.
640 WASH 6000.
650 WAIT.
660 COMMENT***BEGIN STOP REAGENT ADDITION***.
670 FOR I=0 TO N-1 STEP 20.
680 ASPIRATE 10000 OF DILUENT.
690 FOR J=I TO I+19.
700 DISPENSE 500 TO S:RACKUP(J+1).
710 NEXT J.
720 NEXT I.
730 COMMENT***TURN SOLVENT SELECT MVP TO WASH SOLUTION***.
740 ACTUATE DEVcLP02R.
750 COMMENT***RINSE SYSTEM WITH WASH SOLUTION***.
760 WASH 10000.
770 END.
```

*Figure 3. Example of FLEXPREP instruction set for Ohmicron RaPID assay on the Microlab 2200.*

were dynamically extracted for 30 min under the following conditions: carbon dioxide with 10% methanol modifier at 80°C, under 450 atm, at a flow rate of 500 ml/min collected into 5·0 ml of water at pH 4·5. The collection volume was then brought up to 10 ml total volume with distilled water. Six hundred microlitres of the 10 ppb nominal extracts were diluted with 1400 µl of distilled water to yield a final extract concentration, assuming 100% recovery, of 0·9 ppb. Four hundred microlitres of the 50 ppb nominal extracts were diluted with 1600 µl of distilled water to yield a final extract concentration, assuming 100% recovery, of 3.0 ppb. Fifty microlitres of the 500 ppb nominal extracts were diluted with 1950 µl of distilled water to yield a final extract concentration, assuming 100% recovery, of 3·75 ppb.

### *Quantix microplate assays on the Microlab ATplus*

The Hamilton Microlab ATplus is an established automated clinical pipettor used in blood banks and clinical reference laboratories around the world. A schematic of the work area of the ATplus is shown in figure 4; it uses up to 12 disposable positive displacement tips with a 300 µl capacity. These tips can be spread from 9 mm to 20 mm for transfers between test tubes and microplates. The instrument incorporates an automatic tip replenishment magazine and used tip waste receptacle; tips can also be washed by repetitive pumping in a continuously refreshed wash area. Tips can be wiped through prepunched non-wicking paper to remove residual fluid form the outside surface. Ninety-six sample tubes can be accessed in either the 8 or 12 row dimension. In addition, the ATplus has a variety of racks for common laboratory disposables, including microplates, microtubes, test tubes and reagent troughs.

Four soils (two sandy loam and two clay loam) were measured into soil extraction tubes using a soil scoop and extracted into methanol using a soil extraction kit (Quantix, Cinnaminson, NJ, USA, cat. No. SEK-10). Five tubes were prepared for each soil, for a total of 20 tubes. These tubes were then spiked with various levels of alachlor and metolachlor using a 1000 ppb spiking solution (see table 5). One sample of each soil was left unspiked as a control. The extracts were diluted 1:10 with distilled water and analysed by Quantix kits for alachlor (cat. No. ALA MW 2.0) and metalochlor (cat. No. MET MW 2.0).

The extracts were placed on the sample side of the instrument and the reagents; Conjugate, Substrate, and Stop were placed in separate reagent containers in the reagent area. A typical pipetting sequence for these procedures is as follows:

(1) Add 180 µl of diluent (distilled water) to all sample wells of the assay plate.
(2) Pipette 200 µl of calibration standards in duplicate into the assay plate.
(3) Transfer 20 µl of methanol sample extract into the sample wells.
(4) Wash tips five times with 300 µl of isotonic saline between samples.
(5) Add 50 µl of Conjugate to all wells.
(6) Incubate at RT for 10 min.
(7) Wash plate five times with Wash buffer.
(8) Add 200 µl of Substrate to all wells.
(9) Incubate at RT with shaking for 10 min.
(10) Add 50 µl of Stop solution to all wells.

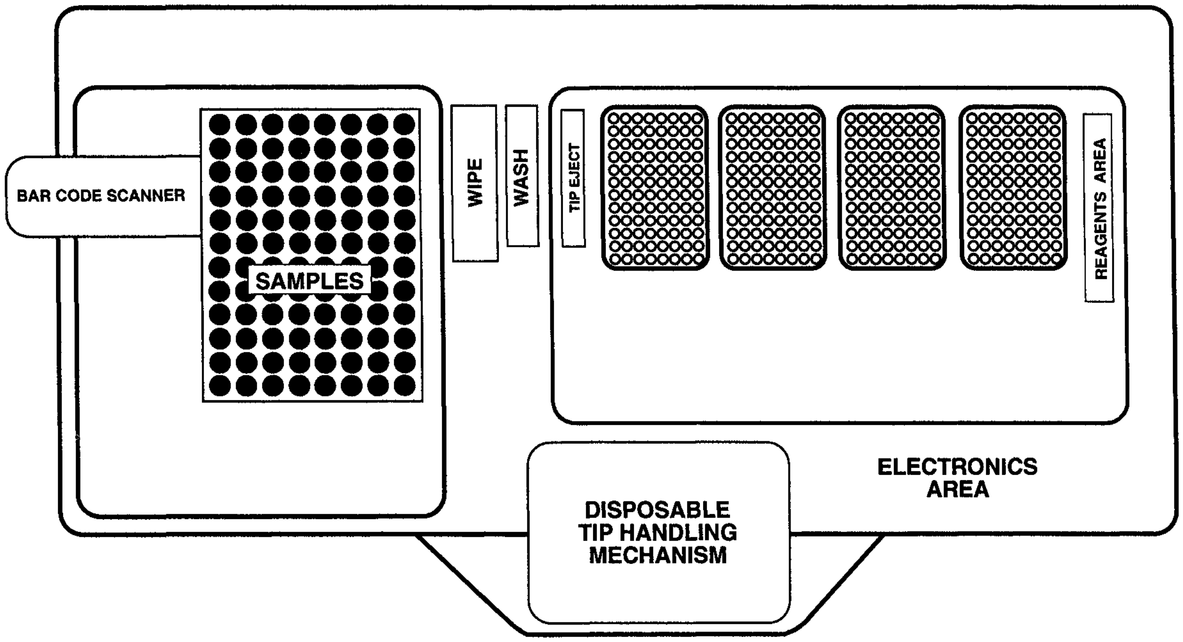

*Figure 4. Diagram of the Microlab ATplus deck.*

(11) Shake plate for 10 s.

(12) Read plate at 630 nm.

An example of the command sequence in SUNPLUS is shown in figure 5.

Following pipetting on the ATplus, the plates were further processed manually using a Quantix Manual Strip Washer System (cat. No. MSW-8) and Quantix Plate Shaker (cat. No. ASP-2). Plates were read on a Biotek EL312 (Winooski, VT, USA) reader at 630 nm.

*Quantix microplate assays on the Microlab F.A.M.E.*

The Hamilton Microlab F.A.M.E. (Fully Automated Microplate Elisa) is a microplate workstation, which, following sample addition to the plates, automates all aspects of ELISA procedures; F.A.M.E. has a modular design as shown in figure 6. The standard configuration includes four modules.

*Entry/incubator module*

In this module, barcoded microplates are loaded and identified. This module also holds two incubation zones, each holding five microplates in individual incubator slots. One of the towers is held at ambient, while the other is programmable to 45°C.

*Incubator module*

This module contains two additional temperature zones with five slots each. These are independently programmable to 70°C.

*Washer/dispenser module*

A 24-channel manifold is used to wash plates with up to three wash solutions and one rinse solution for cleaning the washing head. Each of the manifold's channels is independently controlled to guarantee washing quality and performance. To achieve zero reagent carry-over, a disposable system was developed for reagent dispensing. Eight reagents can be placed in a carousel of dedicated containers with their own individual dispensing syringes. Reagent identification is achieved by bar-coding, and liquid-level detection ensures pipetting integrity.

*Dispenser/photometer module*

A second reagent carousel in this module increases the reagent capacity to 16. Photometric plate measurements are performed using eight available interference filters (340–750 nm). The photometer is suitable for all well types and can be used for endpoint, well scan, and kinetic measurements. Finished plates are also transported to an exit stack in this module. Here they are accessible for further processing or discarding.

*Continuous processing*

A transport system which carries microplates between modules, enabling parallel processing within different modules. An OS/2 based user interface controls module function, procedure scheduling, and photometric data evaluation. The F.A.M.E. processing sequence for module scheduling is seen in figure 7.

### Results and discussion

*Ohmicron RaPID tube assays on the Microlab 2200*

During the development of the protocol for the Ohmicron assay, it became evident that two manipulations were critical to the success of the assay. Initial results for the blanks were up to 20% low and it was discovered that

| No. | Command | Plate | Row | Volume | Mix Vol | Mix Cyc | Height |
|---|---|---|---|---|---|---|---|
| 1 | ********************SAMPLE ADDITION************************ | | | | | | |
| 2 | new Tips........... | | | Tips : • • O O O O O O • • • • | | | |
| 3 | Aspirate Reag. ˜Off | | 2 | 180.0 | | | 30.0 |
| 4 | Tip wipe........... | | | | | | 10.0 |
| 5 | Dispense in Plate.. | 1 | A | 180.0 | 0.0 | 0 | 6.5 |
| 6 | Aspirate Reag. ˜Off | | 2 | 180.0 | | | 30.0 |
| 7 | Tip wipe........... | | | | | | 10.0 |
| 8 | Dispense in Plate.. | 1 | B | 180.0 | 0.0 | 0 | 6.5 |
| 9 | new Tips........... | | | Tips : O O • • • • • • • • • • | | | |
| 10 | Aspirate Sample ˜On | | A | 200.0 | | | 0.0 |
| 11 | Tip wipe........... | | | | | | 10.0 |
| 12 | Dispense in Plate.. | 1 | A | 200.0 | 0.0 | 0 | 5.9 |
| 13 | Aspirate Sample ˜On | | A | 200.0 | | | 0.0 |
| 14 | Tip wipe........... | | | | | | 10.0 |
| 15 | Dispense in Plate.. | 1 | B | 200.0 | 0.0 | 0 | 5.9 |
| 16 | Tip wash........... | | | | | 5 | 10.0 |
| 17 | Tip wipe........... | | | | | | 10.0 |
| 18 | new Tips........... | | | Tips : • • O O O O O O • • • • | | | |
| 19 | Aspirate Sample ˜Off. | | A | 20.0 | | | 43.0 |
| 20 | Tip wipe........... | | | | | | 10.0 |
| 21 | Dispense in Plate.. | 1 | A | 20.0 | 0.0 | 0 | 5.9 |
| 22 | Aspirate Sample ˜Off | | A | 20.0 | | | 43.0 |
| 23 | Tip wipe........... | | | | | | 10.0 |
| 24 | Dispense in Plate.. | 1 | B | 20.0 | 0.0 | 0 | 5.9 |
| 25 | new Tips........... | | | Tips : O O O O O O O • • • • • | | | |
| 26 | Aspirate Reag. ˜On | | 1 | 100.0 | | | |
| 27 | Tip wipe........... | | | | | | 10.0 |
| 28 | Dispense in Plate.. | 1 | A | 50.0 | 0.0 | 0 | 4.0 |
| 29 | Dispense in Plate.. | 1 | B | 50.0 | 0.0 | 0 | 4.0 |
| 30 | ********************SHAKE AND INCUBATE************************ | | | | | | |
| 31 | ********************SUBSTRATE ADDITION************************ | | | | | | |
| 32 | Aspirate Reag. ˜On | | 4 | 200.0 | | | |
| 33 | Dispense in Plate.. | 1 | A | 200.0 | 0.0 | 0 | 4.0 |
| 34 | Aspirate Reag. ˜On | | 4 | 200.0 | | | |
| 35 | Dispense in Plate.. | 1 | B | 200.0 | 0.0 | 0 | 4.0 |
| 36 | ********************SHAKE AND INCUBATE************************ | | | | | | |
| 37 | ********************STOP SOLUTION****************************** | | | | | | |
| 38 | Aspirate Reag. ˜On | | 3 | 50.0 | | | |
| 39 | Dispense in Plate.. | 1 | A | 50.0 | 0.0 | 0 | 4.0 |
| 40 | Aspirate Reag. ˜On | | 3 | 50.0 | | | |
| 41 | Dispense in Plate.. | 1 | B | 50.0 | 0.0 | 0 | 4.0 |

*Figure 5. Example of the SUNPLUS instruction set for the Quantix alachlor assay on the Microlab ATplus.*

some of the magnetic particles were being removed from the walls of the tubes during the washing procedure. This was caused by the probe position and vacuum flow. If the untapered vacuum probe was not centred within the tube, beads would be aspirated to waste, therefore decreasing colour development and giving an elevated background reading. The strength of the vacuum also appeared to be important. At lower vacuum levels the probe had to be held at the bottom of the tube while the fluid level fell and a flow pattern was set up in which the particles were entrained and aspirated to waste. The optimal condition was a stronger vacuum with a fast probe diving speed. This evacuated the tubes quickly, with the probe always remaining just below the receding liquid level. Flow patterns appeared to be directly towards the centre of the tube, rather than a shearing pattern down the walls to the bottom.

Another important aspect in all timed assays is consistent incubation times for the first and last samples of a rack or batch: 'front-to-back' times. In this assay, the reaction of analyte and conjugate with the antibodies bound to the beads begins when the beads are added to the tubes. Unfortunately, the end mark of this competitive reaction

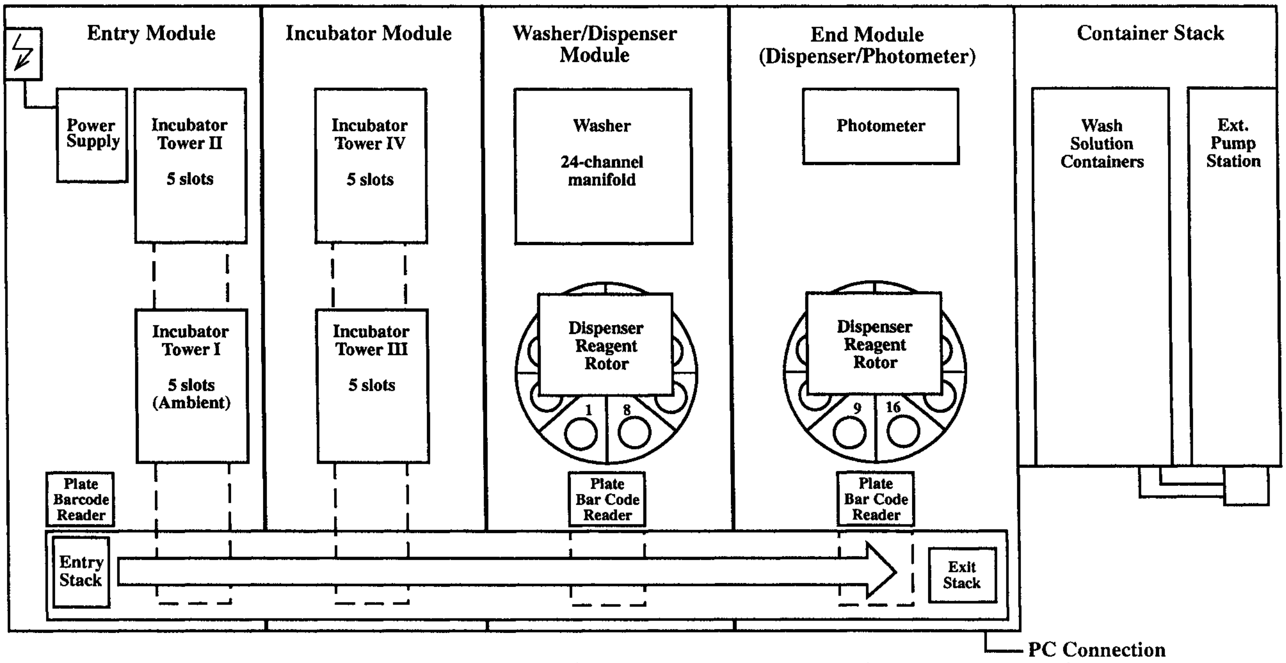

*Figure 6. Schematic of Microlab F.A.M.E. showing entry/incubator, incubator, washer/dispenser, dispenser/photometer modules, and transport system.*

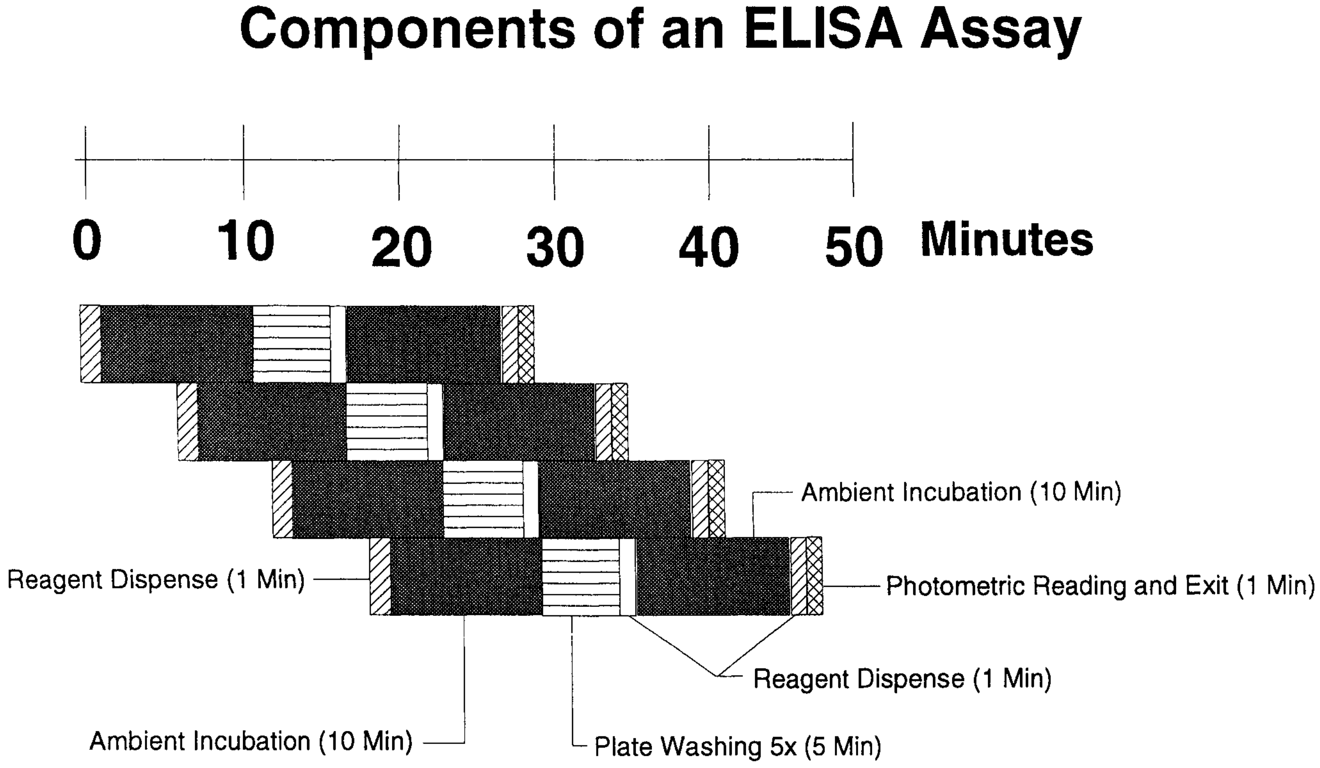

*Figure 7. Scheduling for the Quantix alachlor/metolachlor assays on the Microlab F.A.M.E.*

is when the assay tubes are lowered into the magnetic base; an event which occurs simultaneously for all tubes. This requires the addition of the magnetic beads to be done as quickly as possible; however, if it was too fast, foaming occurred which produced high sample to sample variations. In this work, the delay from first tube to last was only 2 min, with a total incubation time of 20 min; so there was a worst case difference of 10% between first and last sample. In reality, the lowering of the tubes into the magnetic base does not stop the competition altogether and the subsequent evacuation after a 2 min equilibration was a more definitive stopping point. The delay between evacuation of the first tube and the last tube was 2 min 12 s and thus the effective difference in incubation was much less than calculated, approaching a negligible effect. This can be seen from a comparison of the absorbance value in a 3·0 ppb control run in tube No. 9 (0·405 O.D.) and the same control run in tube No. 60 (0·408 O.D.).

*Table 1. Results for SFE-IA of Atrazine spiked soil samples on the Microlab 2200 with Ohmicron RaPID assay.*

| Sample | Spike level (ppb) | Percent recovery | % RSD ($N = 8$) |
|---|---|---|---|
| System blank | | na | na |
| Matrix blank | | na | na |
| Spiked topsoil | 10 | 109 | 3·5 |
| Spiked clay | 10 | 120 | 3·5 |
| Spiked topsoil | 50 | 106 | 2·3 |
| Spiked topsoil | 500 | 118 | 3·5 |

Fortunately, the addition of the Chromogen and Stop are matched exactly in delay from front to back due to equal volumes and identical syringe use. If the volumes had been different, syringe speeds and delays could be manipulated to match the final incubations of all assay tubes. Results for the extracts are shown in table 1.

Carry-over was evaluated by alternating standards and blanks. These assays are meant to be used as screens. Regulatory and litigatory requirements demand false positives over false negatives and measurement bias must be on the high side; the results are shown in table 2. Although carry-over existed to a small extent with the highest standards, any samples reported immediately after an out of range high sample would most likely be retested.

Overall, 60 samples were processed in 1 h 22 min.

*Quantix microplate assays on the Microlab ATplus*

The alachlor standards curve was run with five replicates on the ATplus and manually in a side by side comparison. Precision was excellent for both (see table 3). The time required for the ATplus to pipette this assay (excluding incubations) was 8 min 23 s, while the expert manually pipetted assay was only slightly slower at 8 min 55 s.

The Quantix assays for alachlor and metolachlor both advertise 0·7% cross reactivity for each other. This selectivity was tested by making various mixed standards and analysing for each individually in a single plate frame. Because Quantix offers individual, coated eight well strips for each assay, these were mixed within a single plate frame. Rows 1, 2, 5, 6, 9, and 10 were alachlor while the remaining were metolachlor strips; the results are shown in table 4.

Dilutional rangefinding refers to the automatic serial dilution of samples over, in this case, four orders of magnitude with the hope that one dilution will fall within the calibration range of the assay. This approach could be taken for all samples all of the time, or more likely, on samples whose initial results were shown to be off-scale high values. An intermediate microtube plate was used to prepare the dilutions. Results are shown in table 5. Tips were not discarded and dilutions were done from high to low concentrations which caused carry-over as seen from the overestimation at the higher dilutions.

*Quantix microplate assays on the Microlab F.A.M.E.*

A sample set consisting of standards was pipetted on the ATplus as above and then transferred to the F.A.M.E. for processing and evaluation. The results are shown in table 3.

### Conclusions

This work has demonstrated the benefits of automation for immunoassays. As immunoassays gain acceptance in the analytical laboratory, automation should be embraced by the laboratory technician as a time saving and effective tool. Confirmations may still require chromatographic techniques, followed by selective detectors such as electron capture detectors (ECDs) and mass spectrometry. However, the majority of samples are negative and the screen will obviate the need for an expensive analysis of this group. The sample throughput afforded by these methods, especially when used on automated instruments, will allow much finer resolution in contaminant plume mapping, thus minimizing the cost of remediation. While these procedures were automated on open systems, there will undoubtedly be commercial systems available which are dedicated to a particular group of analytes (probably grouped according to EPA appendices or site characteristics). The monitoring of crops for pesticide and herbicide residues, as well as the exposure of field workers both for entry and overall occupational exposure through body fluids, could allow large-scale studies to be performed at a fraction of the cost of a study based on traditional methods. Sample preparation for immunoassays are much less demanding than for chromatographic methods. Simple filtration of water samples or solvent rinses of soil and produce are often all that is required.

*Table 2. Results of carry-over challenges with Microlab 2200 and Ohmicron RaPID assay. Values represent two blanks following each concentration.*

| Challenge concentration (ppb) | Reading for successive blanks (ppb) | Challenge concentration (ppb) | Reading for successive blanks (ppb) | Challenge concentration (ppb) | Reading for successive blanks (ppb) |
|---|---|---|---|---|---|
| 50 | nd, nd | 500 | 0·05, nd | 5000 | 0·55, 0.10 |
| 50 | 0·17, nd | 500 | nd, nd | 5000 | 0·74, 0·16 |
| 50 | 0·18, 0·19 | 500 | 0·41, 0·10 | 5000 | 0·95, 0·27 |
| 50 | 0·11, 0·15 | 500 | 0·31, 0·28 | 5000 | 0·74, 0·41 |

*Table 3. Precision comparison for manual, ATplus and ATplus/F.A.M.E. processing of Quantix alachlor assay.*

| Control conc. (ppb) | %RSD | | | |
|---|---|---|---|---|
| | Product insert example | Expert manual | ATplus pipetted | ATplus/ F.A.M.E. |
| 0·0 | 4·7 | 1·6 | 1·7 | 4·3 |
| 0·2 | 4·0 | 1·1 | 0·7 | 3·1 |
| 0·5 | 5·9 | 0·9 | 1·4 | 4·9 |
| 1·0 | 3·5 | 1·7 | 0·9 | 5·0 |
| 2·0 | 3·9 | 2·6 | 2·1 | 7·0 |
| 4·0 | 5·2 | 2·4 | 2·6 | 2·2 |
| 8·0 | 5·4 | 3·6 | 2·7 | 6·1 |

*Table 4. Results of mixed spike samples (alachlor and metolachlor) from Quantix alachlor and metolachlor assays.*

| Sample | Conc. (ppb) | | Alachlor | | Metolachlor | |
|---|---|---|---|---|---|---|
| | Ala. | Met. | Separate plates | Mixed plates | Separate plates | Mixed plates |
| 1 | 0 | 0·3 | nd | nd | 0·68 | 0·63 |
| 2 | 0·3 | 1·0 | 0·68 | 0·47 | 1·6 | 1·57 |
| 3 | 1·0 | 4·75 | 1·25 | 1·35 | 6·04 | 5·53 |
| 4 | 4·75 | 0 | 5·8 | 4·97 | nd | nd |
| 5 | 0 | 0·3 | nd | nd | 0·53 | 0·53 |
| 6 | 0·3 | 1·0 | 0·56 | 0·51 | 1·48 | 1·43 |
| 7 | 1·0 | 4·75 | 1·53 | 1·32 | 5·97 | 5·01 |
| 8 | 4·75 | 0 | 6·14 | 5·4 | nd | nd |
| 9 | 0 | 0·3 | nd | nd | 0·80 | 0·70 |
| 10 | 0·3 | 1·0 | 0·86 | 0·80 | 2·31 | 1·92 |
| 11 | 1·0 | 4·75 | 1·25 | 1·35 | 6·04 | 5·53 |
| 12 | 4·75 | 0 | 5·6 | 5·9 | 0·34 | 0·31 |
| 13 | 0 | 0·3 | nd | nd | 0·67 | 0·54 |
| 14 | 0·3 | 1·0 | 0·49 | 0·56 | 1·71 | 1·43 |
| 15 | 1·0 | 4·75 | 1·14 | 1·38 | 5·51 | 4·88 |
| 16 | 4·75 | 0 | 4·9 | 5·04 | nd | nd |

*Table 5. Dilutional rangefinding for Quantix alachlor assay.*

| Conc. (ppb) | Dilution | | | | |
|---|---|---|---|---|---|
| | 1:1 | 1:10 | 1:100 | 1:1000 | 1:10000 |
| 1000 | | | | 1440 | 3300 |
| 100 | | | 106 | 160 | 640 |
| 10 | | 10·1 | 12·5 | 30 | |
| 5·0 | | 5·7 | 7·7 | | |
| 2·0 | 2·45 | 2·3 | | | |
| 1·0 | 1·00 | 1·09 | | | |
| 0·5 | 0·56 | 0·34 | | | |
| 0·1 | 0·16 | | | | |

The ability to perform automatic sample preparations, such as solid phase extraction, filtration, and dilutions, greatly enhance the utility of automated systems. Error-free processing avoids the tedium and confusion associated with large sample batches. Using these techniques, residue analysis could reach the efficiency and throughput found in clinical reference laboratories.

### References

1. Van Emon, J. M. and Lopez-Avila, V., *Analytical Chemistry*, **64** (1992), 79A.
2. Hammock, B. D. and Mumma, R. O., in *Pesticide Analytical Methodology*, Zweig, G. (Ed.) ACS Symposium Series No. 136 (American Chemical Society, Washington D.C., 1980), 321.
3. Mumma, R. O. and Brady, J. F., in *Pesticide Science and Biotechnology*, Greenhalgh, R. and Roberts, T. R. (Eds.) (Blackwell, Oxford, 1986), 341.
4. Lopez-Avila, V., Charan, C. and Beckert, W. F., *Trends in Analytical Chemistry*, **13** (1994), 118.

